# Triaxial accelerometer-measured physical activity and functional behaviours among people with High Grade Glioma: The BrainWear Study

**DOI:** 10.1371/journal.pone.0285399

**Published:** 2023-05-24

**Authors:** Seema Dadhania, Lillie Pakzad-Shahabi, Sanjay Mistry, Matt Williams

**Affiliations:** 1 Computational Oncology Group, Institute of Global Health Innovation, Imperial College London, London, United Kingdom; 2 Radiotherapy Department, Charing Cross Hospital, Imperial College Healthcare NHS Trust, London, United Kingdom; 3 Faculty of Medicine, Department of Surgery and Cancer, Imperial College London, London, United Kingdom; 4 NIHR Clinical Research Department, Medical Oncology, Charing Cross Hospital, Imperial College Healthcare NHS Trust, London, United Kingdom; Pennington Biomedical Research Center, UNITED STATES

## Abstract

**Background:**

High-grade gliomas (HGG) account for 60–75% of all adult gliomas. The complexity of treatment, recovery and survivorship creates a need for novel monitoring approaches. Accurate assessment of physical function plays a vital role in clinical evaluation. Digital wearable tools could help us address unmet needs by offering unique advantages such as scale, cost and continuous real-world objective data. We present data from 42 patients enrolled into the BrainWear study.

**Methods:**

An AX3 accelerometer was worn by patients from diagnosis or at recurrence. Age-, sex-matched UK Biobank control groups were chosen for comparison.

**Results:**

80% of data were categorised as high-quality demonstrating acceptability. Remote, passive monitoring identifies moderate activity reduces both during a course of radiotherapy (69 to 16 minutes/day) and at the time of progressive disease assessed by MRI (72 to 52 minutes/day). Mean acceleration (m*g)* and time spent walking daily (h/day) correlated positively with the global health quality of life and physical functioning scores and inversely with the fatigue score. Healthy controls walked on average 2.91h/day compared to 1.32h/day for the HGG group on weekdays and 0.91h/day on the weekend. The HGG cohort slept for longer on weekends (11.6h/day) than weekdays (11.2h/day) compared to healthy controls (8.9h/day).

**Conclusion:**

Wrist-worn accelerometers are acceptable and longitudinal studies feasible. HGG patients receiving a course of radiotherapy reduce their moderate activity by 4-fold and are at least half as active as healthy controls at baseline. Remote monitoring can provide a more informed and objective understanding of patient activity levels to help optimise health related quality of life (HRQoL) among a patient cohort with an extremely limited lifespan.

## Introduction

World Health Organisation (WHO) Grade III and IV classified high-grade gliomas (HGG) account for 60–75% of all adult gliomas [1, 2]. They are highly heterogenous tumours, arising from glial cells in the central nervous system of which WHO Grade IV glioblastoma multiforme (GBM) is the most predominant and aggressive with a median survival time of 15 months with treatment [3]. Current standard treatment for GBM includes maximal safe surgical resection, radiotherapy (RT) and chemotherapy with temozolomide. The diagnosis of HGG and impact of multimodal treatment options can be associated with functional decline, cognitive impairment, and a deteriorating psycho-social well-being [4, 5]. These have a negative impact on health-related quality of life (HRQoL) which represents the patient’s general perception of the effect of the illness and treatment on physical, psychological, and social aspects of life [6–10]. Limited lifespan of patients with HGG and impact of both tumour and treatment shift the scope of care beyond survival towards optimising functional outcomes and improving HRQoL. Functional and HRQoL outcomes can be evaluated with patient reported outcomes (PROs) which capture the patients’ subjective effects of illness and treatment. They are broadly defined as “any report of the status of a patient’s health condition that comes directly from the patient, without interpretation of the patient’s response by a clinician or anyone else” [11, 12]. There is emerging evidence that PROs are a useful tool in oncology to enhance patient-physician communication as well as improving symptom control and treatment response monitoring. Recent studies have also reported greater benefits of PRO monitoring including improved overall survival and reduced healthcare service utilisation which are contributing toward the paradigm shift of patient-centred care [13].

Within the field of neuro-oncology, functional status, health behaviours and PROs have a significant impact on brain tumour prognosis [14]. Eastern Cooperative Oncology Group (ECOG) Performance Status (PS) (Table 1), a scale widely used in oncology to evaluate patient functional status is a prognostic measure of survival in brain tumours along with age, histological diagnosis, comorbidity, number of prior progressions, tumour localisation [15–22]. A decline in PS at 2 weeks post-operatively has shown to have significant impact on overall survival in patients having maximal surgical debulking [23, 24]. A recent systematic review of physical activity (PA) and exercise in adults diagnosed with primary brain tumours noted that most adults with brain cancer were not sufficiently active from diagnosis through to treatment completion and that high levels of PA were associated with lower severity of brain tumour specific concerns and higher quality of life (QOL). In addition to functional status, fatigue has a detrimental effect on HRQoL and is a strong independent predictor of survival in recurrent HGG [25]. Fatigue is an extremely distressing symptom among patients with brain tumours, and the pathophysiology of cancer-related fatigue in the context of brain tumours is complex involving behavioural, biological, social, and medical factors. Up to 33% of patients with GBM experience Grade 2 and 3 fatigue after radiotherapy though fatigue is often addressed as a complication of treatment thereby failing to acknowledge the impact of the HGG itself [26–29]. A better understanding of HGG related functional status, PA and fatigue levels could guide clinicians in optimising HRQoL and symptom management among a patient cohort with an extremely limited lifespan and may have implications on survival as well [23].

**Table 1:** Eastern cooperative oncology group performance status scale.

Grade	Description
0	Fully active, able to carry on all pre-disease performance without restriction
1	Restricted in physically strenuous activity but ambulatory and able to carry out work of a light or sedentary nature e.g., light housework
2	Ambulatory and capable of all selfcare but unable to carry out any work activities. Up and about more than 50% of waking hours
3	Capable of only limited selfcare, confined to bed or chair more than 50% of waking hours
4	Completely disabled. Cannot care on any selfcare. Totally confined to bed or chair
5	Dead

Measuring cancer patient functional status is currently done using the clinician graded PS scale and PRO questionnaires. Although widely used in oncology practice, there remain several limitations associated with the PS scale. The intermittent nature of evaluating PS at clinical visits only makes the objective assessment of physical functioning challenging given the dynamic nature of a patient’s functional status through their cancer journey. In addition, PS scales are prone to physician bias in that PS can be either under- or over-estimated and are influenced by patient recall bias whereby patients may inaccurately report their past activity or symptom profile. Finally, there remain inaccuracies in the consistency of reporting PS between providers and patients [30]. Clinician-rated functional status does not capture all facets of patient physical function or HRQoL and PROs such as the European Organization for the Research and Treatment of Cancer Quality of Life Questionnaire (EORTC QLQC30) can offer further understanding into patient activity [31]. 

Recent advances in wearable activity monitors and sensing technology have made it possible to gather real-time, objective activity data in a non-obtrusive manner. Remote monitoring can capture changes in behaviour that may reflect meaningful variation in functional status, symptom burden, QOL and other adverse outcomes such as emergency admissions or unscheduled outpatient appointments [32, 33]. Real-time monitoring between clinical encounters can extend the reach of care and provide actionable insights to drive more personalized and proactive care. Although there remain challenges with implementing data from remote monitoring into clinical service delivery, they offer a potentially low cost and non-invasive solution to evaluating the effects of the disease and treatments on patient functioning and daily activities. Triaxial accelerometers consist of small sensors which register acceleration along three axes, and are worn on varying locations of the body, commonly the hip, wrist, and thigh. The term ‘wearable accelerometer’ is often used to describe a wearable device worn to capture PA measures remotely. The raw acceleration data collected from triaxial accelerometry represent the direction and magnitude of acceleration from each axes in the unit *g*, where 1*g* is equivalent to the gravity of earth [34]. The raw data is processed into activity summaries such as step count, calories, activity count and activity classification using developed algorithms and can be represented at varying temporal resolutions e.g., second, minute, hourly, daily [35]

Remote monitoring data can complement snapshots of health gathered during clinical visits and via PRO generated health data. Measuring PROs systematically and between provider appointments reflects a growing appreciation of the ability of PROs to capture symptoms and treatment toxicities, improve communication, enhance QOL and potentially improve survival [13, 36]. Similarly, the objective measure of PA and functional behaviours such as sleep, walking and time spent in moderate activity may enhance our understanding of the effects of cancer and its treatment on daily life, support clinical decision making and act as a triage adjunct for earlier detection of deteriorating health and thus earlier intervention.

We present findings from 42 patients with HGG enrolled into the BrainWear study (ISRCTN34351424), a pilot study evaluating wrist worn accelerometers to capture activity data in patients with primary and secondary brain tumours [35]. It seeks to collect multi-modal longitudinal PROs and clinical data at the point of diagnosis or recurrence and evaluate whether triaxial accelerometer data can be used to explain, influence and/or predict health-related outcomes. Here we present (1) quantitative descriptions of activity at baseline, through radiotherapy and at the time of progressive disease (2) the relationship between activity and PRO measured health outcomes (3) quantitative descriptions of how activity levels and functional behaviours from a UK Biobank cohort of healthy individuals and those with a non-neoplastic brain pathology differ from HGG patients at baseline.

## Materials and methods

### BrainWear study design

**Fig 1:**
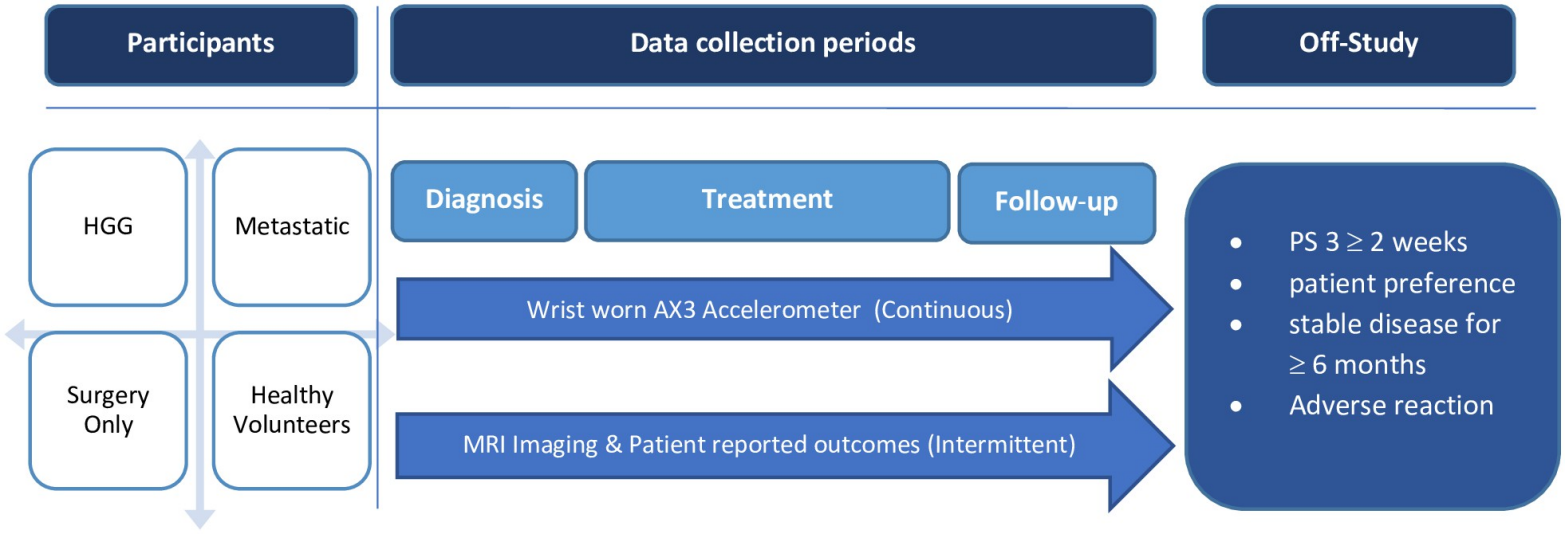
BrainWear trial design. Four categories of participants were eligible for enrolment; (1) HGG—Radiological or histological diagnosis of HGG at diagnosis or recurrence; (2) Surgery Only–Low grade glioma having surgery only; (3) Metastatic–patients with brain metastases fit to have radical treatment (surgery or stereotactic radiotherapy) with >6 months lapsed since previous treatment for brain metastases; (4) Healthy Volunteers–carer of patient taking part in study.

South West–Cornwall & Plymouth Research Ethics Committee (18/SW/0136) approved the study. Between October 2018 and February 2021, patients provided informed, written consent and were recruited into the BrainWear study. Fig 1 shows the trial design. 42 patients recruited into the HGG strata are presented in this analysis. Patients with a radiological diagnosis of HGG were screened via the neuro-oncology MDT and approached for recruitment at early clinical visits. Recruitment into the study was either at the point of suspected (pre-surgery) or histologically confirmed (post-surgery) diagnosis of HGG, or at disease recurrence following primary treatment. At baseline assessment, all participants were provided with an Axivity AX3 wrist worn accelerometer to wear and requested to complete four PRO measures. PROs were collected prospectively at pre-specified study time points and radiology and electronic health record (EHR) data gathered retrospectively. Patients withdrew from the study if they no longer wished to take part, experienced an adverse reaction to the device e.g., skin irritation, if they reached ECOG PS 3 for more than 2 weeks or had radiological evidence of stable disease for more than 6 months.

### Measurements

#### Accelerometer measured physical activity and functional behaviours

For objective evaluation of PA and functional behaviours, we used the Axivity AX3 triaxial accelerometer, a commercial version of the Open Movement AX3 open-source sensor designed by Open Lab, Newcastle University (https://github.com/digitalinteraction/openmovement) and used in the UK Biobank 7-day accelerometer study [37]. The Axivity AX3 accelerometer recorded data at 100Hz with a dynamic range of 8g and has been validated using free-living energy expenditure methods [38]. Patients were asked to wear the device on their non-dominant wrist continuously or for as long as they felt comfortable, with a new device provided at 14-day intervals by postage or collected face-to-face. Upon receipt of returned AX3 device all devices were initialised, and raw data downloaded in raw.cwa format using OmGui (OmGui Version 1.0.0.30, Open Movement, Newcastle UK) and charged for repeated usage.

#### Accelerometer data preparation

Accelerometer data in raw.cwa format, either derived from the BrainWear study participants or a matched UK Biobank control were processed using the UK Biobank accelerometer analysis package available at https://github.com/activityMonitoring/biobankAccelerometerAnalysis [39]. To ensure different AX3 accelerometers provide a similar output under similar conditions, the acceleration signals were calibrated to local gravity using a method described and run in the UK Biobank github (v4.2.2) accelerometer analysis package [40, 41]. Data quality from each accelerometer file was categorised as ‘high’ or ‘low’ based on ≥72 hours of data in a 7-day data collection and data in each 1-hour period of a 24-hour cycle over multiple days. Only ‘high’ quality data was included in the final analysis. Missing data was imputed using the averaged data from similar times of the day. Overall measure of PA is represented as average vector magnitude in milligravity (*mg*) units by calculating the Euclidean norm minus one (ENMO), where higher levels of vector magnitude represent greater activity. To calculate ENMO, the sample level Euclidean norm of the acceleration in the x/y/z axes was calculated and machine noise removed using a fourth order Butterworth low pass filter with 20Hz cut-off filter. One gravitational unit was removed from the vector magnitude to separate out the activity related component of the acceleration signal and remaining values truncated to zero [37]. To classify activity into 5 accelerometer-predicted functional behaviours (sleep, sedentary, light-tasks, moderate and walking) we used the two-stage machine-learning walmsley-2020 model built into the UK Biobank package consisting of balanced random forests and hidden markov models. A 126-dimensional feature vector was extracted for every non-overlapping 30-second time window maintaining the average vector magnitude value over the epoch. This model has shown 88% accuracy in classifying functional behaviours using data from a free-living study of 152 participants who wore an AX3 on their wrist whilst also wearing a camera and completing a sleep diary [42]. The machine-learning model for behaviour classification utilises a smaller set of rotation-invariant features and is designed to be insensitive to how the watch is rotated on the wrist, helpful if participants misorientate the device when removed or replaced during the course of the study Functional behaviours are described in METs (Metabolic Equivalent of Task), which measure energy expenditure relative to energy expenditure in quiet sitting and defined as [42]:

1. Sleep: non-waking behaviour.
2. Sedentary behaviour: waking behaviour of <1.5 MET’s in a sitting, lying or reclining posture.
3. Light activity: waking behaviour at <3 METs not meeting the criteria for sedentary behaviour.
4. Moderate activity: all behaviour ≥3 METs
5. Walking

#### PRO questionnaires

PRO questionnaires selected were the EORTC QLQ-C30 and the brain tumour specific module BN20, the Montreal Cognitive Assessment (MoCA) and the Multidimensional fatigue inventory (MFI) scale. The EORTC QLQ C30 and BN20 measure health and quality in life in brain tumour patients and contain both functional and symptom scales for physical functioning and fatigue. The EORTC QLQ-C30 comprises 30 single questions, 24 of which are aggregated into five functioning scales (physical, role, emotional, cognitive, social), three symptom scales (fatigue, nausea and vomiting, pain) and one global health status scale. The remaining 6 single item scales assess symptoms (dyspnoea, insomnia, appetite loss, constipation, diarrhoea, financial difficulties). The EORTC QLQ BN20 is a 20 question QOL assessment specifically for patients with brain neoplasms. It comprises 11 symptom scales, scored as four multi-item scales (future uncertainty, visual disorder, motor dysfunction and communication deficit) and seven single item symptom scales (headache, seizures, drowsiness, hair loss, itchy skin, weakness of legs and bladder control) and was designed to be used with the core questionnaire QLQ-C30. The self-assessed scale scores from 1–4 are then linearly converted to a 0–100 scale, with severity of symptoms indicated by a higher score. In the case of functions, a higher score is indicative of better function [31, 43, 44]. The MFI is a 20-dimensional scale designed to evaluate five dimensions of fatigue: general fatigue, physical fatigue, reduced motivation, reduced activity, and mental fatigue and has been validated in cancer patients [45]. The MoCA is a rapid screening instrument for mild cognitive dysfunction and evaluates different cognitive domains: attention and concentration, executive functions, memory, language, visuo-constructional skills, conceptual thinking, calculations, and orientation [46]. At study baseline and at each predetermined timepoint, patients were invited to complete PRO questionnaires and the treating oncologist to grade PS according the ECOG PS scale which ranks functional status on a scale of 0 to 5 (Table 1) [38]

#### Clinical data

Medical history and relevant clinical information including MRI imaging, MRI reports, histopathology, radiotherapy and chemotherapy treatment plans and steroid prescriptions were extracted from the patient electronic health record.

### Patient subgrouping

We present data in 4 subgroups described below. Accelerometer data completeness was dependent on patient wear-time during the study. Each subgroup contains a different number of patients dependent on whether the clinical event or outcome measure had paired high-quality accelerometer data within the specified time period thus patients may overlay across the 4 subgroups (Table 2).

Group 1 (Radiotherapy): Patients having RT whilst on-study; accelerometer data required between Day 1 of RT and within 14 days of RT completion.
Group 2 MRI head: Patients having an MRI head whilst on study; accelerometer data required 42 days either side of the date of MRI head with at least 50% of data during this time-period.
Group 3 QOL PRO: Patients completing an EORTC QLQ-C30 and BN20 questionnaire whilst on study; accelerometer data required 14 days either side of the completed questionnaire.
Group 4 HGG case at baseline for matching with UK Biobank controls: Patients at baseline having radiotherapy; accelerometer data required 14 days either side of the date of day 1 RT.

**Table 2:** Description of the 4 subgroups and accelerometer data requirements.

Group	Description	Clinical event or outcome	Paired accelerometer data required	Date range of accelerometer data required	No. patients with paired high-quality accelerometer data
**1**	Course of RT	Patients having RT	Yes	Between Day 1- and 14 days post completion of RT	17
**2**	MRI Brain	Patients having an MRI Brain	Yes	42 days either side of date of MRI	10
**3**	PRO questionnaire	Patients completing an EORTC QLQ-C30 and BN20 questionnaire	Yes	14 days either side of date of questionnaire	24
**4**	HGG case at baseline for matching with UK Biobank controls	Patients having RT	Yes	14 days either side of first day of RT course	19

#### UK Biobank control groups

HRQoL is reported as being significantly impaired at baseline in patients with HGG [25]. For post-hoc exploratory analysis, we identified 2 comparison groups from the UK Biobank large scale population 7-day assessment of physical activity using wrist worn accelerometers, by selecting accelerometer data from up to five age and sex matched controls [37]. To quantify the effects of HGG at baseline and prior to the impact of multimodal treatments, control groups were selected to draw out descriptive differences in PA and functional behaviours in a healthy and unhealthy cohort with a non-neoplastic cerebrovascular disease pathology. We identified the first group self-reporting in the UK Biobank study as ‘apparently healthy’ declaring no medical conditions and in ‘good’ or ‘excellent’ health and able to walk at a subjectively ‘steady’ or ‘brisk’ pace. Patients known to have pre-existing cerebrovascular disease and identified as having a stroke or transient ischaemic attack were selected for the second group [International Classification of Diseases, 10^th^ revision (ICD10) codes: I60-64]. Table 3 provides demographic details of the HGG cases and UK Biobank control groups.

**Table 3:** Demographic details of HGG cases and the two control groups selected from the UK biobank 7-day accelerometer study.

Characteristics	HGG Cohort	UK Biobank Controls	
		Apparently healthy	Prior stroke or TIA
**n**	19	95	95
**Age, years (median)**	65	64	66
**Female, n (%)**	7 (37)	35 (37)	35 (37)
**Male, n (%)**	12 (63)	60 (63)	60 (63)
**Accelerometer wear time, median days (length of data capture in days)**	10.78 (14)	6.92 (7)	6.82 (7)

### Statistical methods

Baseline characteristics were summarised as mean, median or n (%). Unadjusted mean accelerometer-measured vector magnitude (m*g*) and accelerometer-predicted functional behaviours in hours/day (walking, tasks-light, moderate, sedentary, sleep) were presented overall, by two age groups, sex, and by RT treatment days (weekdays) versus non-RT treatment days (weekends). Where applicable, these results were also plotted for the two control populations. Longitudinal data analysis to evaluate temporal changes in activity during RT and at the time of progressive disease were analysed using generalised linear mixed effects models (GLMMs) fit by maximum likelihood. GLMMs estimate fixed and random effects and are particularly useful when the dependent variable is not normally distributed but involves repeated measures, since GLMMs can model autocorrelation [47]. Univariable and multivariable multilevel linear mixed models were constructed in which clustering of patients within studies was considered by a random intercept on the study level. In the multivariable models, adjustments were made for age and sex. To evaluate correlation between PRO questionnaire measures and accelerometer assessed PA and functional behaviours, spearman correlation coefficients were calculated. A two-tailed paired t-test was used to evaluate differences in selected PRO measured multi-item scales between participants. Significance was set at *a* = 0.05, but the interpretation of the p-values should be interpreted with caution as the chance of a Type II error rate is higher in small sample sizes. Python v3.7.3 and R v3.6.0 were used for data processing and statistical analyses, respectively.

## Results

Between October 2018 and February 2021, 42 patients with WHO Grade III or IV HGG were recruited. 40 patients were recruited at their primary diagnosis and 2 patients at presentation of recurrence. 33 of the 42 patients had a course of primary RT whilst on study. In total 3458 days of raw accelerometer data were recorded from 42 patients in 347 files (14-days per file) of which 80% (197 files) were classified as high quality. For each 14-day data capture, median (IQR) wear time was 10.78 (7.09–13.23) days. Median (IQR) overall data collection was 49 (28–91) days. Table 4 provides demographic details of all patients as well as by grouped analysis. Median age was 60 years, 38% (16) were female and 62% (22) were male. At enrolment, 14 patients were assessed by the treating clinician as PS 0 and 28 patients as PS 1. 3 patients withdrew from the study for device related issues; 2 felt the device was uncomfortable to wear and 1 patient reported a skin reaction to the strap.

**Table 4:** Demographics table of 42 patients with HGG enrolled into the BrainWear study presented as all patients and by group. See Table 2 for description of groups. C = lomustine (CCNU); PC = procarbazine and lomustine.

Demographics HGG	n (%)				
	All	Group 1	Group 2	Group 3	Group 4
**n**	42 (100)	17 (40)	10 (24)	24 (57)	19 (45)
**Sex**					
** Female**	16 (38)	6 (35)	3 (30)	8 (33)	7 (37)
** Male**	26 (62)	11 (65)	7 (70)	16 (66)	12 (63)
**Age at study onset, years**					
** 18–40**	5 (12)	1 (6)	1 (10)	2 (8)	0 (0)
** 41–60**	15 (36)	4 (24)	2 (20)	8 (32)	6 (32)
** 61–80**	22 (52)	12 (70)	7 (70)	12 (50)	13 (68)
**Grade of disease at diagnosis (WHO)**					
** III**	8 (19)	2 (12)	2 (20)	4 (17)	4 (21)
** IV**	34 (81)	15 (88)	8 (80)	20 (83)	15 (79)
**PATIENTS RECRUITED AT DIAGNOSIS**	40 (95)				19 (100)
**Primary Surgery**					
** Yes**	39 (98)	16 (94)	9 (90)	24 (100)	18 (95)
** No**	1 (2)	1 (6)	1 (10)	0 (0)	1 (5)
**Surgical Procedure**					
** Debulking**	28 (70)	14 (88)	9 (100)	20 (83)	13 (72)
** Biopsy**	11 (30)	2 (12)	0 (0)	4 (17)	5 (28)
**Primary Radiotherapy**					
** Yes**	33 (83)	17 (100)	9 (90)	20 (83)	19 (100)
** No**	7 (17)	0 (0)	1 (10)	4 (17)	0 (0)
**Primary Radiotherapy total dose**					
** 55–60 Gy (6-week course)**	22 (67)	11 (65)	7 (78)	16 (80)	15 (79)
** ≤45 Gy (3-week course)**	11 (33)	4 (35)	2 (12)	4 (20)	4 (21)
**Adjuvant Primary Chemotherapy**					
** Yes**	26 (65)	12 (71)	8 (80)	16 (67)	15 (79)
** No**	14 (35)	5 (29)	2 (20)	8 (33)	4 (21)
**PATIENT’S RECRUITED AT RECURRENCE**	n = 2 (5)	0	0	0	0
**First treatment at relapse**					
** Chemotherapy**	2 (100)	n/a	n/a	n/a	n/a
**Chemotherapy regime**					
** PC or C**	2 (100)	n/a	n/a	n/a	n/a
** Temozolomide**	0 (0)	n/a	n/a	n/a	n/a

Group 1 (Radiotherapy): Patients having RT whilst on-study; accelerometer data required between Day 1 of RT and within 14 days of RT completion.

Group 2 MRI head: Patients having an MRI head whilst on study; accelerometer data required 42 days either side of the date of MRI head with at least 50% of data during this time-period.

Group 3 QOL PRO: Patients completing an EORTC QLQ-C30 and BN20 questionnaire whilst on study; accelerometer data required 14 days either side of the completed questionnaire.

Group 4 HGG case at baseline for matching with UK Biobank controls: Patients at baseline having radiotherapy; accelerometer data required 14 days either side of the date of day 1 RT.

### Group 1: Physical activity and functional behaviours among HGG patients having radiotherapy (n = 17)

Among HGG patients receiving a course of primary RT who provided paired accelerometer data, unadjusted mean accelerometer-measured vector magnitude and accelerometer-predicted functional behaviours were presented. Patients treated with curative intent received a 6-week course of radiotherapy whilst those treated with the intent of palliation or disease-control were given a 3-week course in accordance with guidelines. 33 patients received a course of RT whilst on study, 24 of whom provided at least a single 14-day accelerometer recording during their RT treatment, of which 17 were included for final analysis (Table 2). There were no significant differences in daily vector magnitude or time spent across different functional behaviours between males and females (p>0.05). Overall mean daily activity was 14.27 m*g* in the <60-year-old group and 15.13 m*g* in the ≥60-year-old group. Both groups spent a similar amount of time walking (82 vs 74 minutes/day). There were no significant differences in time spent performing different functional activities or daily vector magnitude when age was taken into consideration in GLMMs (p>0.05). Time spent walking on RT (weekday) versus non-RT days (weekend) was significantly different, with participants walking on average 20 minutes more on RT-days (p = 0.01, 95% CI = -0.021, -0.6127).

**Fig 2:**
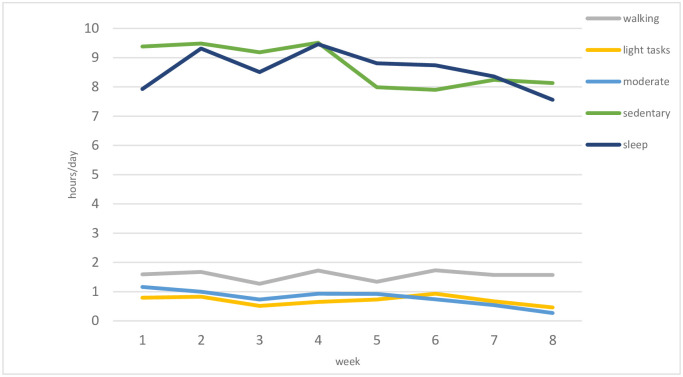
Time spent in each functional behaviour per day in 12 patients who completed a 6-week course of radiotherapy for HGG.

In those patients specifically having a 6-week course of RT (n = 12), time spent in moderate intensity activity fell from 69 to 16 minutes per day between week 1 and the second week following RT completion (Fig 2). GLMMs showed once we account for variation among patients, vector magnitude (m*g)*, moderate, light-tasks and sleeping functional behaviours in h/day negatively correlate with a change over the course of RT which represents increasing daily dose in Gy, from Day 1 to Day 42. Table 5 shows details of accelerometer-measured activity and functional behaviours of the HGG BrainWear cohort, overall and by baseline characteristics during RT. Table 6 shows the mean values and significance test results of 5 multi-item scales scored from the EORTC-QLQ-C30 and BN20 QOL questionnaires at study timepoint pre-RT and post-RT. The questionnaire derived multi-item scales chosen for analysis were based on clinical judgement, with scales selected if they were felt to be clinically linked to activity levels. Physical functioning derived from the EORTC QLQ-C30 and motor dysfunction from the BN20 questionnaire both showed statistically significant differences between the two study timepoints representing a perceived decline in physical ability through a 6-week course of RT. Although the mean fatigue score did increase between the two timepoints, this was not at a statistically significant level.

**Table 5:** Mean accelerometer-measured activity and functional behaviours of the HGG BrainWear cohort, overall and by baseline characteristics.

			Estimate of functional behaviours (h/day)				
Characteristic	n	mean vector magnitude *(*m*g)*	walking	light tasks	moderate intensity	sedentary activity	sleep
**all participants**	17						
**Age, years**							
** 18–60**	6	14.27	1.37	0.52	0.55	9.9	11.64
** >60**	11	15.13	1.24	0.62	0.87	10.39	10.86
**p value**		0.165	0.434	0.493	0.401	0.446	0.434
**95% CI**		-1.54, 0.0002	-0.029, 0.012	-0.069, 0.033	-0.24, 0.096	-0.00089, 0.00039	-0.0009, 0.002
**Sex**							
** M**	12	14.93	1.45	0.52	0.62	9.99	11.41
** F**	5	14.96	1.23	0.66	0.94	10.46	10.69
**p value**		0.921	0.342	0.391	0.662	0.614	0.616
**95% CI**		-1.43, 0.012	-0.52, 0.18	-1.24, 0.49	-2.15, 3.39	-0.00080, 0.013	-0.033, 0.020
**Radiotherapy day (all RT patients)**	17						
** Yes (weekday)**		17.144	1.52	0.67	0.89	9.13	9.09
** No (weekend)**		16.85	1.21	0.60	0.80	8.54	9.19
**p value**		0.0548	**0.0117** *	0.907	0.779	0.830	0.335
**95% CI**		0.0056, 0.00057	-3.84, -0.482	-3.88, 3.44	-3.46, 3.23	-0.107, 0.086	-0.054, 0.158
**Radiotherapy day (6-week course RT only)**	12						
** Yes (weekday)**		18.51	1.66	0.78	0.92	9.07	8.87
** No (weekend)**		16.85	1.35	0.71	0.80	8.40	8.51
**p value**		0.177	**0.0138** *	0.929	0.914	0.295	0.129
**95% CI**		-0.00087, 0.00047	0.319, 2.81	-3.66, 3.34	-3.65, 3.12	-0.049, 0.161	-0.206, 0.026
** Week 1**		19.72	1.59	0.79	1.16	9.38	7.93
** Week 2**		19.29	1.67	0.83	1.00	9.48	9.31
** Week 3**		14.79	1.27	0.51	0.73	9.18	8.51
** Week 4**		17.64	1.72	0.65	0.93	9.51	9.46
** Week 5**		16.48	1.34	0.73	0.92	7.99	8.81
** Week 6**		18.75	1.73	0.93	0.74	7.90	8.74
** Week 7 (post RT)**		15.80	1.57	0.67	0.54	8.24	8.36
** Week 8 (post RT)**		12.12	1.57	0.46	0.27	8.13	7.56
**p value**		**0.00089** *	0.415	**0.0439** *	**0.0087** *	0.076	**0.0028** *
**95% CI**		0.000068, 0.000026	-0.0009, 0.0022	0.00013, 0.0097	0.0016, 0.011	0.000013, 0.00028	-0.0005, -0.0001

* indicates statistical significance (p < .05)

**Table 6:** Mean score of EORTC-QLQC30 and BN20 derived multi-item scales at study timepoint (1) pre-RT and (2) post-RT in 12 participants having a 6-week course of RT. Two-tailed p-value determined using a paired t-test.

PRO scale	Pre-RT	Post RT	p value	95% CI
**fatigue**	37.37373737	43.4343434	0.36743026	-20.36, 8.24
**global QOL**	65.15151515	55.3030303	0.17378283	-5.13, 24.83
**physical functioning**	84.24242424	75.7575758	0.0394*	0.64, 21.17
**future uncertainty**	39.84818182	38.6363636	0.43942647	-16.05, 18.47
**motor dysfunction**	10.1010101	28.2828283	0.0251115*	-33.57, -2.78

*indicates statistical significance; PRO = patient reported outcome; RT = radiotherapy; QOL = quality of life

Fatigue, global QOL and physical functioning are taken from the EORTC QLQ C-30 questionnaire; future uncertainty and motor dysfunction are taken from the BN20 questionnaire.

### Group 2: Physical activity and functional behaviours among HGG patients with progressive disease on MRI (n = 10)

**Fig 3:**
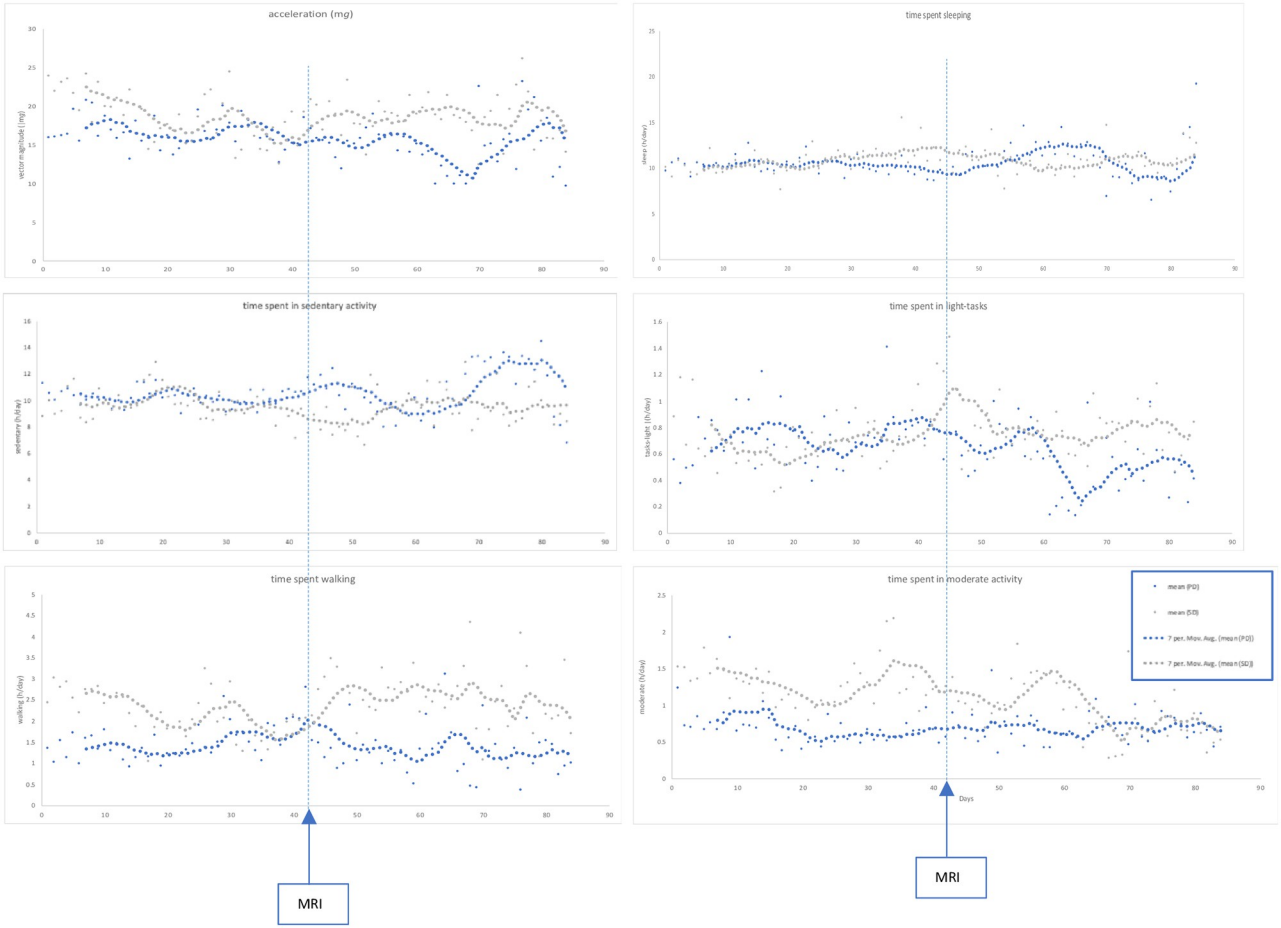
Accelerometer measured acceleration (m*g*) and time spent in functional behaviours (h/day) in the 42 days before and after an MRI scan of the head for disease staging. The date of the MRI is standardised to Day 42. PD = progressive disease; SD = stable disease/disease improvement. Date of MRI scan standardised to Day 42. Dotted lines represent 7-day rolling average.

We analysed accelerometer data in the 42-days before and after an MRI Brain and included participants who provided at least 50% accelerometer data during this time-period (n = 10). Six patients provided accelerometer data on 7 MRI scans which confirmed progressive disease (PD). Four patients had paired data on 4 MRI scans showing stable disease or disease improvement (SD). Demographics are presented in Table 2. GLMMs show time spent doing moderate activity negatively correlated with PD on MRI (p = 0.022, 95% CI 1.015, 1.22). In patients who had either stable disease or disease improvement on their MRI scan, there was no significant difference in time spent in moderate activity (p = 0.463). Fig 3 shows accelerometer measured acceleration (m*g*) and time spent in functional behaviours in hours/day with a 7-day rolling average trendline in the 84-day period surrounding an MRI where the MRI was standardised to Day 42 for analysis.

### Group 3: Correlation between accelerometer- and questionnaire-derived measures of physical activity, fatigue and quality of life (QOL) among HGG patients (n = 24)

**Fig 4:**
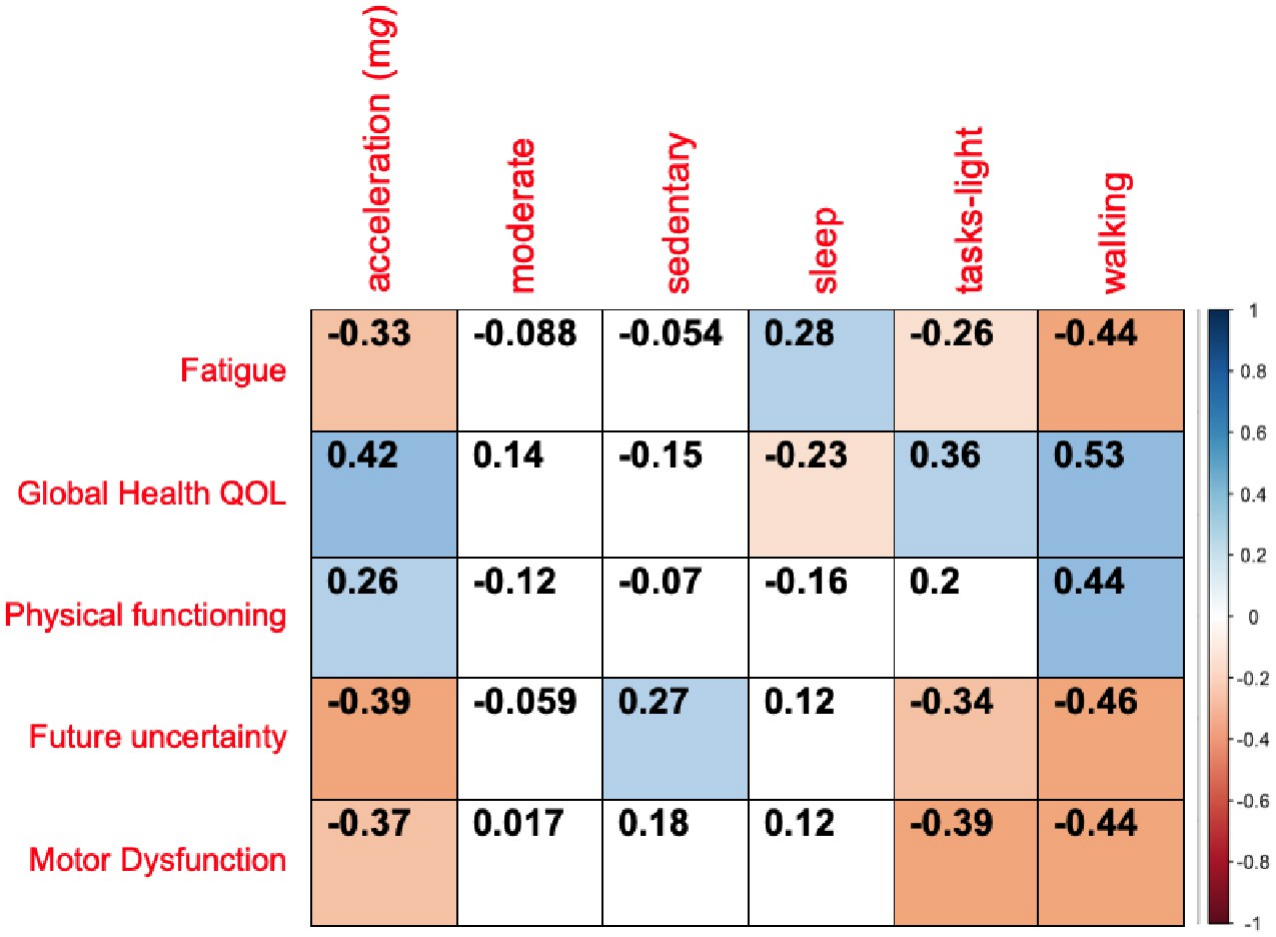
Correlation between accelerometer-measured physical activity and functional behaviours with the EORTC QLQ C30 and BN20 questionnaire results for the HGG patient cohort. Data are Spearman correlation co-efficient.

At each protocol defined timepoint in the study, patients were invited to complete an EORTC QLQ-C30 and BN20 quality of life questionnaire. Correlation analysis was performed between accelerometer and questionnaire-derived measures if patients had contributed accelerometer data within 14 days either side of the completed questionnaire. The questionnaire derived multi-item scales chosen for correlation analysis were based on clinical judgement, with item scales selected if they were felt to be clinically linked to activity levels. The fatigue, global health QOL and physical functioning scales were selected from the EORTC QLQ-C30 and future uncertainty and motor dysfunction from the BN20 questionnaire. 24 patients provided 63 completed questionnaires with paired accelerometer data. Mean acceleration (m*g)* and time spent walking daily (h/day) correlated positively with the global health QOL and physical functioning and inversely with the fatigue score (Fig 4). Motor dysfunction and future uncertainty scales correlated inversely with mean acceleration and time spent walking or carrying out light-tasks.

### Group 4: Comparison of accelerometer-measured physical activity and functional behaviour patterns between HGG patients and UK Biobank control groups at baseline (n = 19)

**Fig 5:**
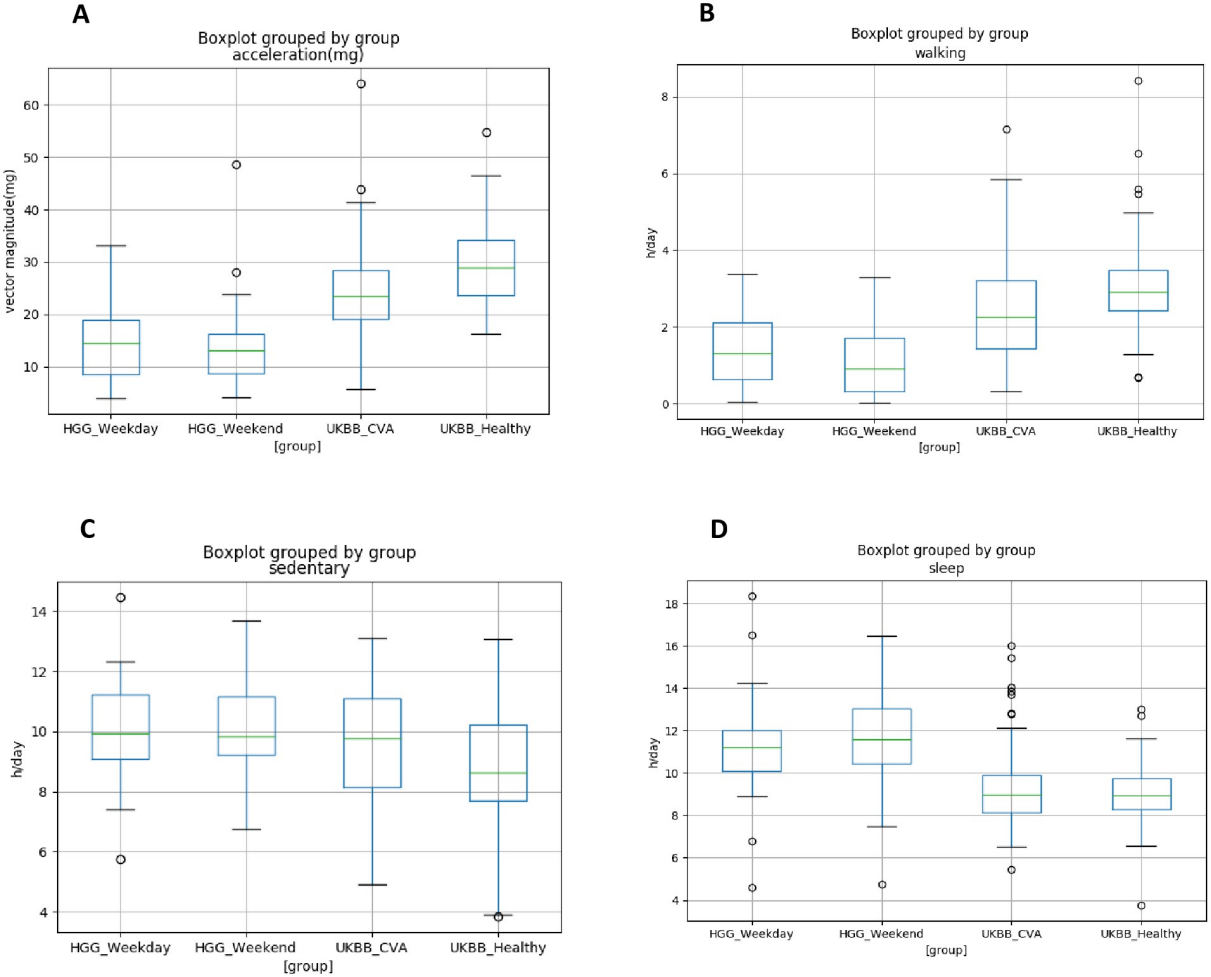
Accelerometer measured vector magnitude (A) and estimates of time spent in functional behaviours in h/day, walking (B), sedentary activity (C) and sleep (D).

**Fig 6:**
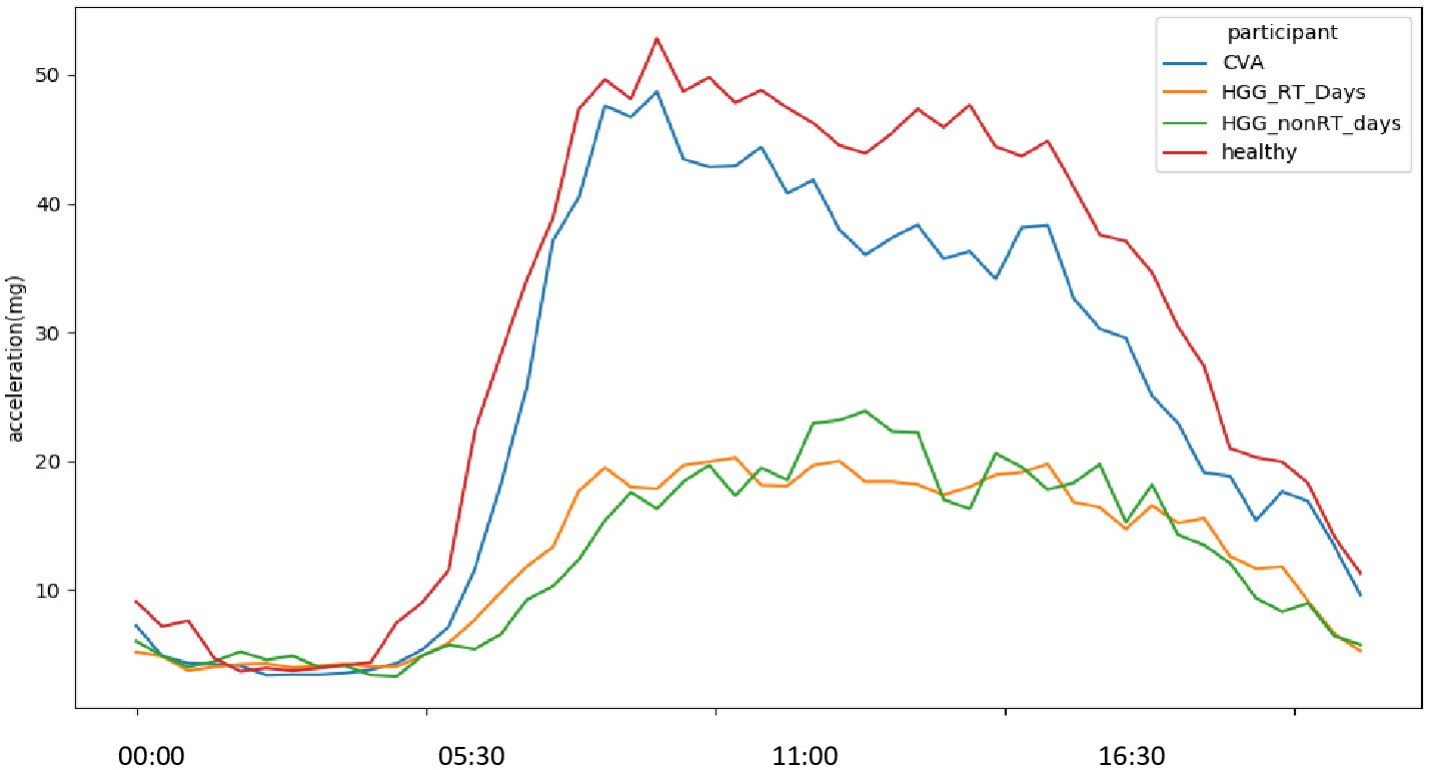
Accelerometer measured average vector magnitude *(*m*g)* according to time of day in HGG patients on RT and non-RT days and two UK Biobank control populations. CVA = cerebrovascular accident.

To understand baseline PA and functional behaviours in patients with a HGG before the effect of multi-modal treatment, we performed analysis at time baseline. We compared this with 2 age and sex matched cohorts from the UK Biobank 7-day accelerometer study; healthy volunteers and participants with a non-neoplastic pathology of the cerebrovascular system to draw out descriptive differences. In HGG cases, we selected a 14-day accelerometer file which fell into a period of 28 days (14 days before and after the first fraction of RT as a comparator to UK Biobank controls. This provided a representation of the patient’s ‘baseline’ activity prior to the side effects of RT. Of the 24 HGG patients who provided high-quality PA data during this period, 5 were excluded for comparison as their age fell out of range of the UK Biobank study (44 to 79 years). PA and functional behaviours in the HGG group were divided across weekdays and weekends as RT is delivered only on weekdays and patients are thought to be active given travel to hospital is required daily for treatment. The HGG group showed lower levels of overall activity compared to the UK Biobank control groups. Mean vector magnitude *(*m*g)* on weekdays for the HGG cohort was 14.56 m*g*, compared with 29 m*g* for apparently healthy controls and 23.45 m*g* for matched controls reporting a previous history of a cerebrovascular accident (CVA). The HGG group were less likely to walk and more likely to take part in sedentary behaviours than apparently healthy matched controls. Apparently healthy controls walked on average 2.91 h/day compared to 1.32 h/day for the HGG group on weekdays and 0.91 h/day on the weekend. The HGG cohort slept for longer on weekends (11.6 h/day) than weekdays (11.2 h/day) compared to matched healthy controls (8.9 h/day) (Fig 5). Fig 6 shows average vector magnitude according to the time of day in the HGG and UKBB control groups.

## Discussion

To our knowledge, this pilot study represents the longest, continuous accelerometer study of a cancer patient population. It provides an objective understanding of functional behaviours and PA levels at baseline as well as the effect on these metrics of RT to the brain. We show that longitudinal accelerometer data collection is feasible and acceptable in a HGG patient cohort.

In the post-operative setting, a 6-week course of RT is offered to patients who have a PS of 0 or 1 and have tumour associated factors which allow for delivery of complex RT with intent of cure. In reality, the 5-year survival of patients with Grade IV GBM receiving maximal treatment is just 5% and disease on average recurs at 6 months to 1 year, so understanding functional status can guide clinicians in optimising QOL and symptom management among a patient group with an extremely limited lifespan [48]. In this study, 12 patients provided accelerometery data whilst receiving a 6-week of RT. Nationally, RT is delivered on weekdays only and requires daily travel to the hospital which can be prolonged by attendance to outpatient clinics or other investigations. Weekend activity is thus likely to be more representative of patient activity hence separated out for analysis. Here we show significant differences in time spent walking between RT and non-RT days, with patients walking up to 20 minutes less on non-RT days. At baseline and prior to the effects of multi-modal treatment, we show HGG patients spend just 54 minutes/day on the weekend walking versus 174 minutes in the apparently healthy UK biobank cohort. Moderate intensity functional behaviours such as brisk walking or general gardening fell significantly from a mean of 96 minutes/day to just 16 minutes/day in the second week following completion of RT when peak toxicity is expected. This corroborated with PRO captured aspects of physical activity (physical functioning, motor dysfunction) which were significantly worse after completion of RT (Table 6). This data provides clinical teams with quantitative estimates of patient functional behaviours in HGG patients considered to be fit enough to have maximal treatment and could act as an adjunct to existing PRO measures. Recovery time to baseline activity level after expected radiotherapy peak may be a useful indicator of coping and help guide clinicians in providing further supportive care to patients felt not to be coping. Similarly, albeit in a small sample number, we have shown time spent in moderate activity reduces at or near the time of progressive disease confirmed by gold-standard MRI. Coomans et al have published a recent analysis of 5539 patients to evaluate factors associated with HRQoL deterioration in glioma patients during the progression-free survival period. They identified only PS was associated with HRQoL deterioration at a statistically significant and clinically relevant level and strengthens the requirement for more robust methods of physical function evaluation to measure HRQoL [49]. Given the well-documented limitations of the PS scoring system objective evaluation of functional capacity in brain tumour patients has so far been limited to tests such as the six-minute walk test (6MWT), or invasive and intermittent investigations such as the cardiopulmonary exercise test (CPET) and body composition measures [50, 51]. The 6MWT has previously been shown to correlate with PS scoring and several QOL domains and equivalent to 56 +/- 13% of that predicted for age and sex [52]. Wearable accelerometers offer more objective and fine-grained activity data and if evaluated in real-time, could have the potential to alert clinical teams of functional behavioural changes consistent with radiation toxicity or progressive disease, and act as an alert system for supporting clinical decision making and optimising HRQoL.

Using statistical methods which take into consideration both the between and across participant variation of repeated measures, we show there are no differences in overall measures of activity or functional behaviours between gender or age. This strengthens our existing approach of evaluating patient fitness for treatment on an individual basis rather than using pre-defined age cut-offs. These findings support a patient centred approach to assist HGG patients through their cancer journey who face both neurological and cancer-treatment related symptoms. We evaluated accelerometery data against HRQoL PRO measures and showed overall daily activity represented as daily vector magnitude *(*m*g)* and time spent walking correlated positively with global health QOL and physical function scores of the EORTC QLQ-C30 questionnaire and inversely with fatigue as well as the motor dysfunction and future uncertainty aspects of the brain-tumour specific module QLQ-BN20. Brain tumour patients experience a considerable level of physical impairment which compromises their QOL and ability to self-care. They experience tumour and treatment related fatigue levels which are in the region of 40–50% more severe than normative levels for cancer patients [50, 52]. RT has been reported to cause early fatigue in up to 80% of patients and can develop into chronic fatigue in up to 30% of patients [53]. Fatigue in HGG is a strong predictor of decreased patient satisfaction and HRQoL and may represent one of the central reasons for discontinuing treatment [54–56]. In clinical practice, the use of accelerometery as a substitute or in addition to these aspects of HRQoL PRO measures may be of benefit in capturing patient HRQoL and has a potential role in improving collection of clinical trial HRQoL outcome measures. Walking is a useful measure of overall health [49, 50] and measuring characteristics of gait such as step count and speed is becoming a robust method of determining varying facets of health [57]. The high granularity of accelerometer data (100hz = 100 readings per second) lends itself to evaluation of more specific elements of walking such as gait speed, which have traditionally required expensive and large laboratory systems e.g. gaitRite [58–60]. Existing algorithms such as the method used in this analysis can characterise functional behaviours including walking. Analysis of peak characteristics such as peak height, width and distance between peaks in short segment epochs e.g., 10 seconds can be used to further characterise gait features. Gait speed has well-documented predictive value for health-related outcomes such as mortality, QOL and physical and cognitive functional decline in older people and will be an area of further work to explore the association between gait features and clinical events such as disease progression, hospitalisation and QOL measures in this dataset [55, 61, 62]. 

Limitations to this study include the use of a machine learning model trained on 152 members of the general population which demonstrated 88% accuracy in correctly classifying functional behaviours. Classification in an unhealthy population is likely to be less accurate, though a study evaluating PA in patients receiving dialysis showed a 74% accuracy when using the same classifier [63]. A further limitation is comparison of HGG accelerometer data which was taken from the non-dominant wrist to that of the UK biobank worn on the dominant wrist. At study design phase, we sought opinion via patient and public involvement sessions. In this case, patients felt wear-time would be higher on the non-dominant hand given the longitudinal nature of the study versus a 7-day collection as per the UK biobank study. Comparability of accelerometer signal aggregation metrics across wrist placement observed PA patterns to be concordant for dominant and non-dominant wrists and statistical parametric mapping analysis did not show statistical differences between the 0–50^th^ percentile of the accelerations produced where 93% of HGG lie. They also showed estimations from previously validated cut-points on the non-dominant wrist and their translation to the dominant wrist were almost equal [64]. Given the longitudinal design of the study, we were restricted by the number of AX3 devices available at any one time thus multiple devices were used across single participants and is a further limiting factor of the study design. Finally, UK Biobank participants may have been subject to the Hawthorne effect resulting in artificially higher activity levels, whilst this may be attenuated over long-term longitudinally monitored populations such as in this study [65].

Strengths of this study include the longitudinal capture of physical activity data with over 80% of accelerometer data classified as high quality and minimal drop-out. This dataset is the first of its kind in linking electronic health record, PRO, and accelerometer data in a HGG cohort, and given the rarity and vulnerability of this population is an invaluable dataset to further our understanding in optimising HRQoL. We advocate the use of research grade accelerometers such as the AX3 which capture activity in a raw format to preserve data integrity. One of the challenges of using wearable devices from differing commercial manufacturers and where raw data is pre-processed remains the concealment of proprietary algorithms and device settings, which can be changed at the manufacturer’s discretion at the time of software updates. A move towards raw sensor data collection in clinical studies provides the opportunity to build large datasets in specific patient groups and increases transparency in clinical research whilst future proofing the data for use with models currently not fully developed.

In conclusion, accelerometery provides a feasible and acceptable way of understanding functional behaviours and PA levels in patients with HGG having active treatment and in the follow-up period. Our analysis highlights the potential role of longitudinal remote monitoring in the clinical setting and begins to inform the clinical and health data community of how passive accelerometer data can be used to support clinical decision making and as an adjunct to PRO measures. Overall daily vector magnitude and time spent walking appear to provide early useful indicators of PA assessment as patients progress through RT and correlate with physical function aspects of well recognised HRQoL PRO questionnaires. Patients with HGG at baseline appear to be less than half as active as age, sex matched healthy control and show lower levels of activity then those with a history of a CVA. Further work may support its use in developing more patient-centred outcomes to evaluate treatment effects and symptom management interventions as well as an alert system prior to events such as hospitalisation or at the point of disease progression.
